# ANCA testing strategies: A single centre experience over the past decade in a large teaching hospital in the Netherlands

**DOI:** 10.1371/journal.pone.0357495

**Published:** 2026-09-09

**Authors:** Maria A. C. Wester Trejo, Tjallingius Martijn Kuijper, Geeke J. Waverijn, Ingeborg M. Bajema, René M. A. van den Dorpel, Marc R. Kok, Iris J. A. M. Verberk-Jonkers, Els J. M. Zirkzee, Snjezana Kos, Annelies E. Berden

**Affiliations:** 1 Department of Pathology, Leiden University Medical Center, Leiden, The Netherlands; 2 Pathology Laboratory, Pathan B.V., Rotterdam, The Netherlands; 3 Department of Rheumatology and Clinical Immunology, Maasstad Hospital, Rotterdam, The Netherlands; 4 Business Intelligence, Maasstad Hospital, Rotterdam, The Netherlands; 5 Department of Pathology and Medical Biology, University Medical Center Groningen, Groningen, The Netherlands; 6 Department of Internal Medicine, Maasstad Hospital, Rotterdam, The Netherlands; 7 Laboratory of Clinical Chemistry, MaasstadLab, Maasstad Hospital, Rotterdam, The Netherlands; 8 Department of Rheumatology, Leiden University Medical Center, Leiden, The Netherlands; University Hospital of Bologna Sant’Orsola-Malpighi Polyclinic Department of Digestive System: Azienda Ospedaliero-Universitaria di Bologna Policlinico Sant’Orsola-Malpighi Dipartimento dell’apparato digerente, ITALY

## Abstract

**Objectives:**

In 2017 a new consensus for antineutrophil cytoplasmic antibody (ANCA) testing was published, based on data from tertiary referral centres for ANCA-associated vasculitis (AAV). AAV-prevalence in their tested population was relatively high. We analysed how the consensus approach performs in a large teaching hospital and tailored it to secondary care settings with lower patient prevalences in their population.

**Methods:**

Patients screened for ANCA between 2012–2023 were included; clinical data were retrospectively collected. Indirect immunofluorescence (IIF) was performed using EUROPLUS^TM^ Granulocyte Mosaic 25 IIF. MPO/PR3 was detected by ImmunoCAP®250. Statistical analyses were performed in *R.*

**Results:**

286/5518 (5.2%) patients tested for ANCA were positive: 63% had no AAV. Non-AAV patients often had other autoimmune diseases, malignancies, infections or used specific drugs. IIF and ELISA as first test showed good negative predictive values. ELISA as first test showed higher positive predictive value and specificity; performing a second test increased specificity. ANCA concentrations were higher in AAV than non-AAV, but ranges were wide.

**Conclusion:**

The majority of ANCA-positive patients did not have AAV. In our secondary care setting, ELISA performs best as first test to *rule out* and to *rule in* AAV. A second test should be considered with high clinical suspicion and a negative first test, or with low antibody concentrations, following the consensus. Additionally, we propose a second test when there is clinical doubt and a positive first test. This increases specificity and PPV, reducing false positives, which is especially relevant in secondary care settings.

## Introduction

ANCA-associated vasculitides (AAV) are systemic small-vessel vasculitides associated with antineutrophil cytoplasmic antibodies (ANCA). Most patients have proteinase 3 (PR3)-ANCA or myeloperoxidase (MPO)-ANCA [1]. AAV is subdivided into microscopic polyangiitis (MPA), granulomatosis with polyangiitis (GPA) and eosinophilic granulomatosis with polyangiitis (EGPA) [2].

ANCA are not specific for AAV and found in other conditions, including other autoimmune diseases, malignancies and infections, and they can be drug-induced. Vasculitis can also occur in many of these conditions, making it difficult to distinguish them from AAV [3,4].

ANCA were discovered in 1959 and associated with vasculitis in 1982 [5–7]. Different detection methods were developed, the most important being indirect immunofluorescence (IIF) and antigen-specific enzyme-linked immunosorbent assays (ELISAs). IIF can show a cytoplasmic (c-ANCA), perinuclear (p-ANCA) or atypical (x-ANCA) pattern. PR3 antibodies are mostly linked to a c-ANCA pattern and MPO to a p-ANCA pattern. IIF can also detect antibodies binding to autoantigens other than MPO and PR3 such as human neutrophil elastase, that can be found in drug-induced AAV [8].

The first international consensus on ANCA testing was published in 1999, providing ANCA screening recommendations when AAV is suspected [9]. The primary screening method was IIF, followed by an immunoassay testing for PR3- or MPO-ANCA in IIF-positive samples. It stated that ideally, both tests should be performed. In 2016, the European Vasculitis Society (EUVAS) performed a multicentre study, evaluating the value of IIF versus antigen-specific immunoassays, showing that the diagnostic use of the latter was equal to, or better than that of IIF [10]. A revised consensus was published in 2017, proposing preferred use of antigen-specific assays for ANCA-screening over IIF. With clinical suspicion of AAV and negative test results, a second antigen-specific test or IIF can be considered. Taking antibody level into account and considering a second test with low antibody levels is also recommended [11].

**Fig 1:**
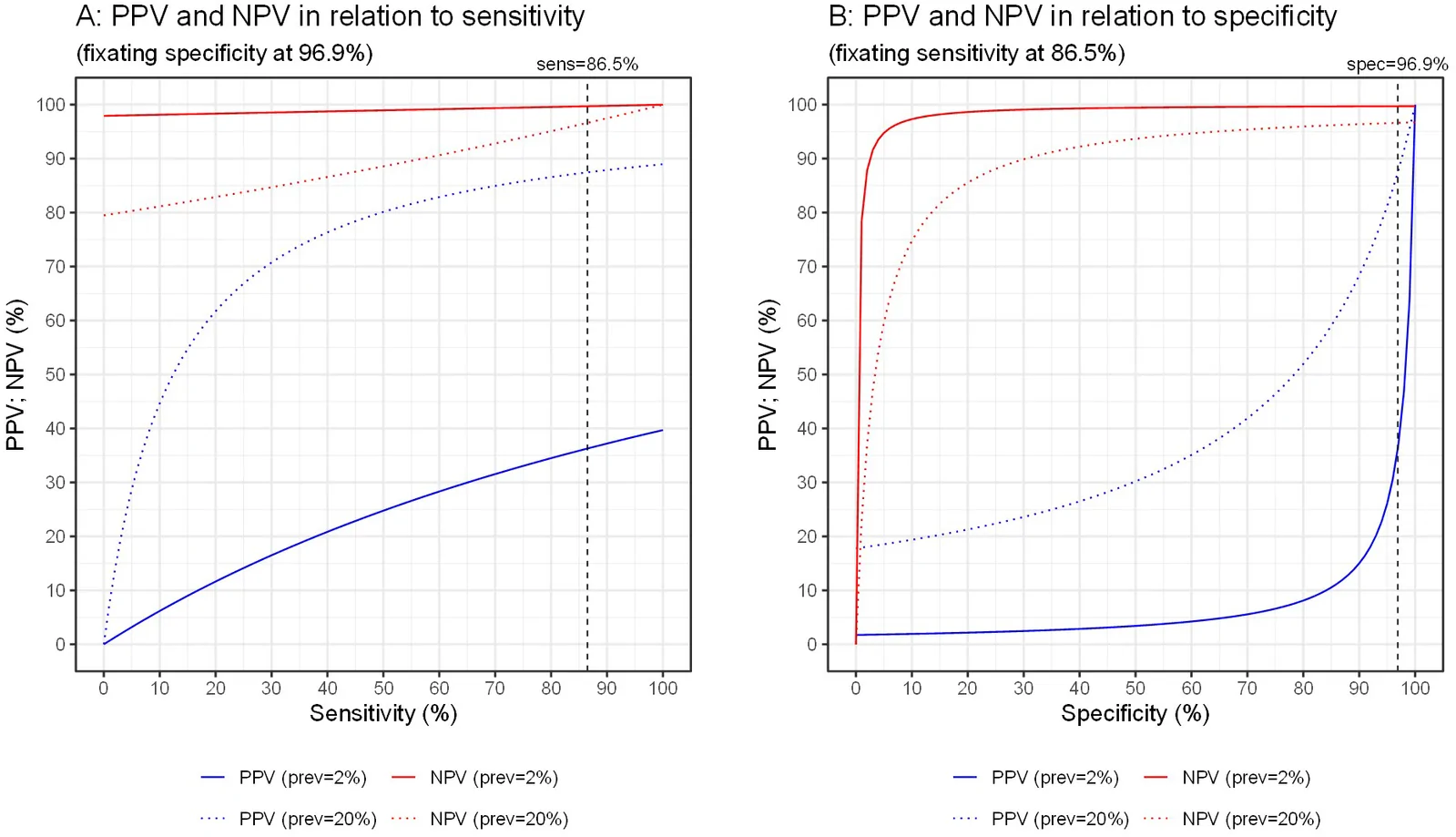
General relationship between test characteristics for settings with different prevalences of AAV patients in the tested population (2% vs 20%). **(A)** Relationship between sensitivity and PPV (blue) and NPV (red); **(B)** relationship between specificity and PPV and NPV. Solid lines represent a 2% prevalence of AAV (approximating our setting), and dotted lines represent 20% prevalence (approximating the EUVAS multicentre setting). Higher prevalence results in higher PPV. At high specificity—such as typically observed for immunoassays measuring PR3 and MPO—the impact of prevalence on PPV is pronounced: with sensitivity fixed at 86.5% and specificity at 96.9%, PPV increases from ~35% at 2% prevalence to ~88% at 20% prevalence (≈50% absolute increase). The EUVAS multicentre study (Damoiseaux *et al.* 2017) tested different immunoassays including the ELISA used in our centre. From their data, we derived the sensitivity (86.5%) and specificity (96.9%) for our ELISA when used as a first-line test. These are indicated by vertical lines. To the right of these lines, increasing test performance shows that in a low-prevalence setting, increasing sensitivity results in only a minimal increase in PPV (A), whereas changes in specificity have a larger effect (B). NPV varies minimally across prevalence levels (<5%). These findings illustrate that while sensitivity and specificity are intrinsic test characteristics, PPV and NPV are strongly influenced by disease prevalence. *Abbreviations*: AAV (ANCA-associated vasculitis), ELISA (enzyme-linked immunosorbent assay), NPV (negative predictive value), PPV (positive predictive value), prev (prevalence).

The 2017 consensus approach was devised using data from tertiary referral centres, where the proportion of AAV patients in the tested population (i.e., the prevalence) is expected to be higher compared to general hospitals. This impacts directly on pre-test probability and positive predictive value of the ANCA test: these are higher in a setting with a high AAV prevalence. Fig 1 shows the general relationship between different patient prevalences in a tested population and test characteristics.

As AAV prevalence in the tested population has direct influence on the diagnostic value of an ANCA test, it is important to evaluate test strategies in settings with different – i.e., lower – prevalences. We therefore assessed the different consensus approaches in a large teaching hospital in the Southwest of the Netherlands over the past decade. Three periods were discerned: a first period following the 1999 consensus, a second period validating automated ANCA read-out and a third period following the 2017 consensus. This study evaluates if the current consensus approach performs equally well in secondary care settings, where the prevalence of AAV is lower than in the tertiary care settings that the consensus was based on. We also take into account the clinician’s objective when requesting the test: is it used for *ruling in* AAV or for *ruling it out*. Finally, we assess the significance of MPO/PR3 concentrations. Combining our findings, we provide an external validation and propose a practical refinement of the 2017 international consensus in a low-prevalence secondary care setting.

## Materials and methods

### Patients and samples

All patients (n = 5518) screened for ANCA in Maasstad Hospital between January 1, 2012 and August 1, 2023 were included in this study. Only diagnostic ANCA test requests, i.e., ‘first tests,’ were used in the analysis. Data were accessed for research purposes on 24-11-2023.

Data were pseudonymised where identifying information was not part of the dataset, but a key to re-identify individuals was available. The study was conducted in accordance with the ethical principles stated in the Declaration of Helsinki.

Data from the electronic medical record (ChipSoft version 6.1) were stored in a SQL-database and extracted using SQL Server Management Studio (version 17.1).

The diagnosis of AAV was manually checked from medical records. All patients fulfilled the ACR 2022 classification criteria for MPA, GPA or EGPA. Comorbidities were extracted using registration of ‘DBC care products’ (*Diagnose Behandel Combinatie* meaning Diagnosis Treatment Combination). The following comorbidities were extracted: malignancy (carcinoma, melanoma, haematological malignancy, sarcoma, glioma), infectious disease (hepatitis B, hepatitis C, lues/syphilis, tuberculosis, HIV, infective endocarditis, other), autoimmune disease (IBD, SLE, RA, sarcoidosis, other) and drug usage (minocycline, thyroid medication, hydralazine, immune suppressants, cocaine). For malignancy, auto-immune and infectious disease, those registered within 5 years before ANCA testing were included. For drug usage, 6 months preceding testing were considered. Registration of malignancies was checked manually to extract specific malignancies for which no DBC care product was present. Because reliable registration of DBC care products is not available for SLE, the diagnosis of SLE was also checked manually. Medication prescriptions were automatically extracted to check for specific drugs. Medication was included if the prescription period overlapped with the moment of ANCA testing.

### ANCA detection methods and testing strategies during different periods

ANCA IIF was performed using EUROPLUS^TM^ Granulocyte Mosaic 25 immunofluorescence (Euroimmun AG, Lübeck, Germany). Evaluation was performed manually by skilled technicians using a Zeiss Axiostar plus fluorescence microscope. When ANCA IIF was positive, ELISA was performed. MPO- and PR3-antibodies were detected by ImmunoCAP250 (Thermofisher Phadia, Sweden). Results were considered positive when above the upper limit of normal (ULN) of the manufacturer (MPO-ANCA 5 U/mL, PR3-ANCA 3 U/mL).

In the first period (January 1, 2012-December 5, 2020), ANCA testing was performed according to the 1999 consensus [9]: i.e., a positive IIF screening test was followed by ELISA. In the second period (December 5, 2020-November 15, 2021), manual IIF read-out was replaced by automated IIF read-out using Europattern Suite (Euroimmun AG, Lübeck, Germany). The EUROStar III Plus LED fluorescence microscope was used with EUROLabOffice 4.0 software. During this period automated IIF and ELISA were done simultaneously for all patients and positive IIF was not a requirement to perform ELISA. This corresponds with ‘the ideal approach’ of the 1999 consensus. In the third period (November 15, 2021-August 1, 2023), ANCA testing was performed according to the 2017 consensus [11]. ELISA was performed as screening test. A second test - IIF - could be ordered upon clinicians’ request.

During the entire study period IIF and ELISA were performed under stable laboratory conditions using commercial tests from the same manufacturers. The only difference in the testing methods themselves is that from period 2 onwards the read-out of IIF was automated.

### PR3- or MPO-ANCA concentrations

Using all ELISA results obtained over the periods, the distribution of PR3- or MPO-ANCA concentrations in patients with and without AAV was analysed and graphically demonstrated using violin plots.

Throughout the text we have stated ‘ANCA-positivity’ when this was the final result of the test strategy that was used. When ANCA-positivity was in fact ‘MPO/PR3 positivity’ by ELISA this is stated explicitly.

### Statistics

Baseline characteristics for AAV and non-AAV patients were described and compared using simple descriptive statistics. Continuous variables were tested using student’s t-test or Wilcoxon ranksum test if distribution was skewed. Categorical variables were tested using Pearson chi-squared test or Fisher’s exact test. Diagnostic performance measures sensitivity (Sen), specificity (Spe), positive predictive value (PPV) and negative predictive value (NPV) were calculated; 95% confidence intervals (CI) were obtained by the Wilson score method implemented in the ‘binconf’ function [12]. Positive (LR+) and negative (LR-) likelihood ratios were calculated, 95% confidence limits were calculated by the Wald/Katz-log method implemented in the ‘BinomRatioCI’ function [13]. All analyses were performed using *R* version 4.2.2. [14].

## Results

### Patients

**Fig 2:**
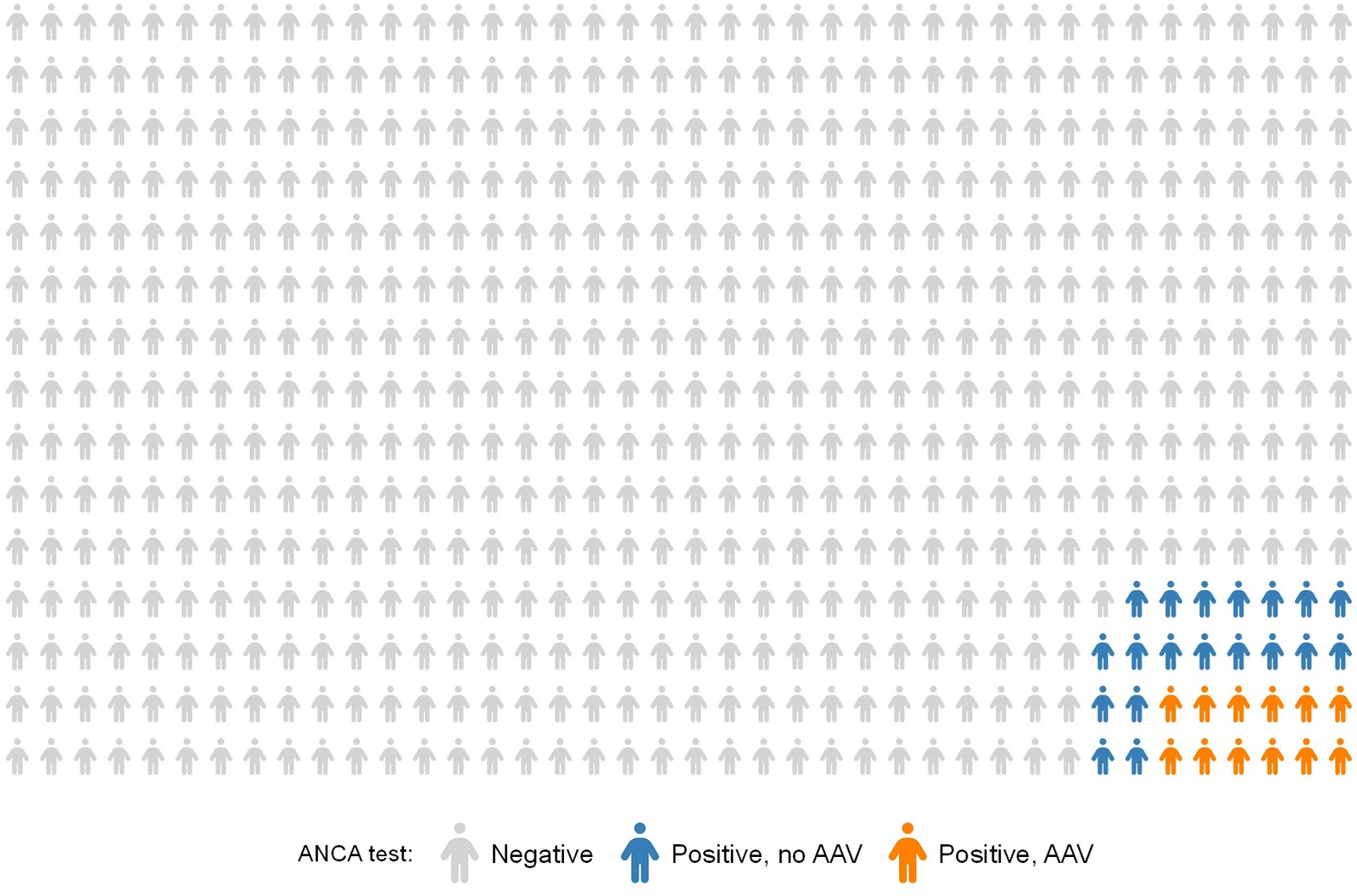
People graph visualizing the numbers of ANCA-positive patients with and without AAV in the tested population. A total of 5518 patients were tested for ANCA, of whom 286 (5.2%) tested positive. Of those, 107 (37.4%) were diagnosed with AAV (orange) and 179 (62.6%) had a positive ANCA test without having AAV (blue). *Abbreviations*: AAV (ANCA-associated vasculitis), ANCA (antineutrophil cytoplasmic antibodies).

5518 patients were tested for ANCA and 286 (5.2%) were positive. Of these, 107 patients (37%) were diagnosed with AAV, meaning that 1.9% of the tested population had AAV. This is shown graphically in Fig 2. In the different periods the prevalences were 1.9.% (period 1), 1.2% (period 2) and 2.5% (period 3). Of the 107 ANCA-positive AAV patients, 52 (48.6%) had MPO-ANCA and 55 (51.4%) PR3-ANCA.

In our cohort, the majority of ANCA-positive patients (63%) did not have AAV. Table 1 summarizes clinical characteristics of ANCA-positive patients without AAV. Of those patients, 51 (28.5%) had another autoimmune disease, 30 (16.8%) a malignancy, 18 (10.1%) an infection and 60 (33.5%) used drugs associated with presence of ANCA. Infections included Hepatitis B/C, HIV, syphilis and tuberculosis. Malignancies included mainly carcinomas, but also haematological malignancies, sarcomas and melanomas. Of patients with other autoimmune diseases 22 (12.3%) had IBD, 18 (10.1%) RA and 7 (3.9%) SLE. Strikingly, five patients had a clinical diagnosis of RA before getting diagnosed with AAV. These patients all presented with arthritis years before onset of vasculitis. Two of these patients had positive RF, but all were anti-CCP antibody negative. It cannot be ruled out that arthritis was the first presenting feature of AAV in these patients.

**Table 1:** Characteristics of patients with a positive ANCA test, with and without vasculitis.

	Total (n = 286)	No vasculitis (n = 179)	Vasculitis (n = 107)	P-value
**Period, n (%)**				0.023^c^
1. Consensus 1999	226 (79.0)	146 (81.6)	80 (74.8)	
2. Internal validation	23 (8.0)	17 (9.5)	6 (5.6)	
3. Consensus 2017	37 (12.9)	16 (8.9)	21 (19.6)	
**Female, n (%)**	144 (50.3)	94 (52.5)	50 (46.7)	0.410^c^
**ANCA, n (%)**				0.065^f^
MPO-ANCA	156 (54.5)	104 (58.1)	52 (48.6)	
PR3-ANCA	126 (44.1)	71 (39.7)	55 (51.4)	
Double positive	4 (1.4)	4 (2.2)	0 (0.0)	
Negative	0 (0.0)	0 (0.0)	0 (0.0)	
**Age, mean (SD)**	60.5 (17.7)	59.7 (18.6)	62.0 (16.1)	0.275^t^
Malignancy, n (%)*	48 (16.8)	30 (16.8)	18 (16.8)	1.000^c^
Carcinoma, n (%)	35 (12.2)	22 (12.3)	13 (12.1)	1.000^c^
Melanoma, n (%)	1 (0.3)	0 (0.0)	1 (0.9)	0.374^f^
Haematological malignancy, n (%)	15 (5.2)	10 (5.6)	5 (4.7)	0.951^c^
Sarcoma, n (%)	1 (0.3)	1 (0.6)	0 (0.0)	1.000^f^
Glioma, n (%)	1 (0.3)	1 (0.6)	0 (0.0)	1.000^f^
Infectious diseases, n (%)*	21 (7.3)	18 (10.1)	3 (2.8)	0.021^f^
Hepatitis B, n (%)	5 (1.7)	4 (2.2)	1 (0.9)	0.654^f^
Hepatitis C, n (%)	1 (0.3)	1 (0.6)	0 (0.0)	1.000^f^
Lues/syphilis, n (%)	5 (1.7)	4 (2.2)	1 (0.9)	0.654^f^
Tuberculosis, n (%)	2 (0.7)	1 (0.6)	1 (0.9)	1.000^f^
HIV, n (%)	2 (0.7)	2 (1.1)	0 (0.0)	0.530^f^
Other infections, n (%)	6 (2.1)	6 (3.4)	0 (0.0)	0.087^f^
Infective endocarditis, n (%)	1 (0.3)	1 (0.6)	0 (0.0)	1.000^f^
Autoimmune disease, n (%)*	57 (19.9)	51 (28.5)	6 (5.6)	<0.001^c^
RA, n (%)	23 (8.0)	18 (10.1)	5 (4.7)	0.163^c^
SLE, n (%)	7 (2.4)	7 (3.9)	0 (0.0)	0.048^f^
Sarcoidosis, n (%)	4 (1.4)	4 (2.2)	0 (0.0)	0.301^f^
IBD, n (%)	23 (8.0)	22 (12.3)	1 (0.9)	0.001^c^
Other autoimmune disease, n (%)	11 (3.8)	11 (6.1)	0 (0.0)	0.008^f^
Drugs usage, n (%)**	103 (36.0)	60 (33.5)	43 (40.2)	0.313^c^
Minocycline, n (%)	1 (0.3)	1 (0.6)	0 (0.0)	1.000^f^
Thyroid medication, n (%)	1 (0.3)	1 (0.6)	0 (0.0)	1.000^f^
Hydralazine, n (%)	0 (0.0)	0 (0.0)	0 (0.0)	N/A
Immune suppressants, n (%)	96 (33.6)	55 (30.7)	41 (38.3)	0.236^c^
Cocaine, n (%)	7 (2.4)	5 (2.8)	2 (1.9)	1.000^f^

*Number of patients with at least one registration of malignancy, infectious disease or autoimmune disease occurring within 5 years before ANCA testing: more than one comorbidity could be registered in an individual patient.

** With regard to drug usage, the 6 months preceding testing were taken into account.

*Abbreviations*: ANCA (antineutrophil cytoplasmic antibodies), HIV (human immunodeficiency virus), IBD (inflammatory bowel disease), MPO (myeloperoxidase), n (number), N/A (not applicable), PR3 (proteinase 3), RA (rheumatoid arthritis), SD (standard deviation), SLE (systemic lupus erythematosus).

^c^Pearson’s chi-squared test.

^f^Fisher’s exact test.

^t^Student’s t-test.

### Test results over the different periods

During period 1, the 1999 consensus was followed and 3846 manual IIF tests were performed. Considering only the IIF results (Table 2), sensitivity was 92.0% (95%CI 83.6–96.3), specificity 96.9% (95%CI 96.3–97.4), PPV 37.3% (95%CI 30.7–44.5), NPV 99.8% (95%CI 99.6–99.89) and LR + 29.9 (95%CI 24.7–36.). IIF followed by ELISA led to an increase in PPV to 76.2% (95%CI 66.1–84.0) and LR+ to 162.6 (95%CI 104.1–254.1).

**Table 2:** ANCA test strategies in the different testing periods.

	Period 1 (2012–2020)						
	N	Sen (95% CI)	Spe (95% CI)	PPV (95% CI)	NPV (95% CI)	LR+ (95% CI)	LR- (95% CI)
**IIF as first test**	3846	92.0 (83.6–96.3)	96.9 (96.3–97.4)	37.3 (30.7–44.5)	99.8 (99.6–99.9)	29.9 (24.7–36.2)	0.08 (0.04–0.18)
**IIF + ELISA**	3835	86.5 (76.9–92.5)	99.5 (99.2–99.7)	76.2 (66.1–84.0)	99.7 (99.5–99.9)	162.6 (104.1–254.1)	0.14 (0.08–0.24.2)
	**Period 2 (2020–2021)**						
**IIF + ELISA**	366	83.3 (43.6–99.1)	98.6 (96.8–99.4)	50.0 (23.7–76.3)	99.7 (98.4–100.0)	60.0 (23.4–153.8)	0.17 (0.03–1.0)
	**Period 3 (2021–2023)**						
**ELISA**	829	100.0 (84.5–100.0)	98.5 (97.4–99.1)	63.6 (46.6–77.8)	100.0 (99.5–100.0)	67.3 (38.4–118.1)	0 (0–0.37)

*Abbreviations*: ANCA (antineutrophil cytoplasmic antibodies), ELISA (enzyme-linked immunosorbent assay), IIF (indirect immunofluorescence), LR+ (positive likelihood ratio), LR- (negative likelihood ratio), N (number), NPV (negative predictive value), PPV (positive predictive value), Sen (sensitivity), Spe (specificity), 95% CI (95% confidence interval).

In period 2, automated IIF was validated and 366 patients were tested with both automated IIF and ELISA. Specificity and NPV were high, as in period 1. However, due to the limited number of AAV cases, confidence intervals for sensitivity, PPV and LR+ were wide (Table 2).

In period 3, the 2017 consensus was followed and 829 patients were tested with ELISA. No additional IIF tests were requested. Sensitivity (100%, 95%CI 0.85–1) and specificity (98.5%, 95%CI 0.97–0.99) values were high, PPV was 63.6% (95%CI 46.7–77.8) and LR + 67.3 (95%CI 38.4–118.1).

Table 3 shows that, when comparing the first step in screening (IIF in period 1 and ELISA in period 3), ELISA as a first test shows a significantly higher PPV (63.6 vs 37.3) and LR+ (67.3 vs 29.9) than IIF as a first test (*p* = 0.008 and *p* = 0.039 respectively). However, when IIF is followed by ELISA as a second test, specificity increases significantly (99.5%; *p* = 0.007) compared to ELISA alone. Also, a significant increase in LR+ (162.6; *p* = 0.047) is observed when two tests are performed.

**Table 3:** Test periods compared.

IIF as 1^st^ test vs ELISA	IIF as 1^st^ test (period 1)		ELISA (period 3)		*P*-value
	Estimate	95% CI	Estimate	95% CI	
**Sen**	0.920	(0.836–0.963)	1.000	(0.845–1.000)	0.334
**Spe**	0.969	(0.963–0.974)	0.985	(0.974–0.991)	**0.018**
**PPV**	0.373	(0.307–0.445)	0.636	(0.466–0.778)	**0.008**
**NPV**	0.998	(0.996–0.999)	1.000	(0.995–1.000)	0.365
**LR+**	29.9	(29.9–36.2)	67.3	(38.4–118.1)	**0.039**
**LR-**	0.083	(0.038–0.178)	0.000	(0.000–0.374)	1.000
**IIF+ELISA vs ELISA**	**IIF + ELISA (period 1)**		**ELISA (period 3)**		***P*–value**
	**Estimate**	**95% CI**	**Estimate**	**95% CI**	
**Sen**	0.864	(0.769–0.925)	1.000	(0.845–1.000)	0.111
**Spe**	0.995	(0.992–0.997)	0.985	(0.974–0.991)	**0.007**
**PPV**	0.762	(0.661–0.840)	0.636	(0.466–0.778)	0.254
**NPV**	0.997	(0.995–0.999)	1.000	(0.995–1.000)	0.227
**LR+**	162.6	(104.1–254.1)	67.3	(38.4–118.1)	**0.047**
**LR-**	0.136	(0.076–0.242)	0.000	(0.000–0.374)	1.000

*Abbreviations*: ELISA (enzyme-linked immunosorbent assay), IIF (indirect immunofluorescence), LR+ (positive likelihood ratio), LR- (negative likelihood ratio), N (number), NPV (negative predictive value), PPV (positive predictive value), Sen (sensitivity), Spe (specificity), 95% CI (95% confidence interval).

### MPO- and PR3-ANCA concentrations

**Fig 3:**
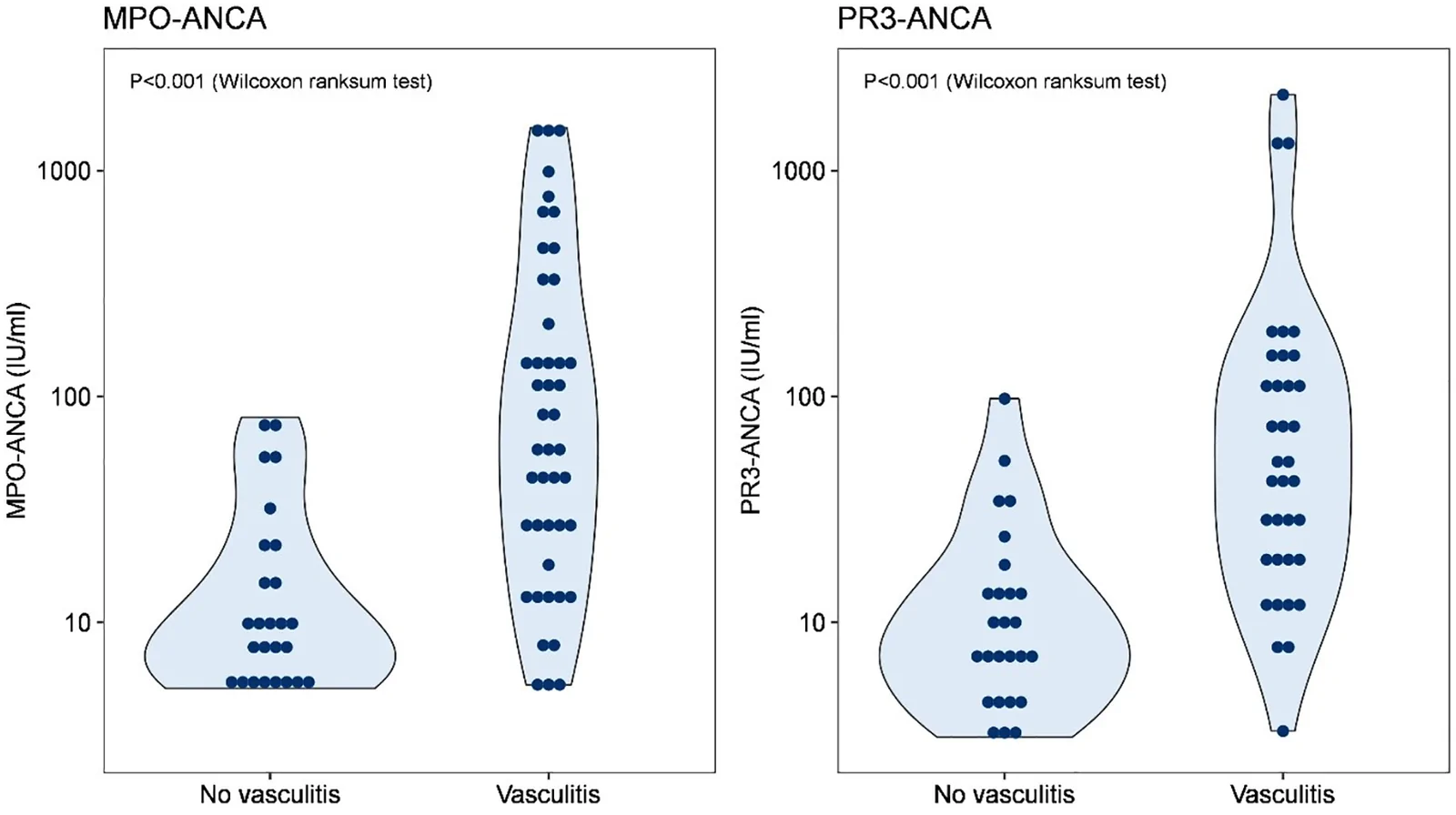
Violin plot showing MPO/PR3 concentrations for MPO/PR3-positive patients with and without ANCA-associated vasculitis. Both MPO-ANCA and PR3-ANCA concentrations were higher for patients with vasculitis than without (*p* < 0.001). For MPO-ANCA, median concentrations were 63 U/mL (IQR 25-210) for patients with AAV versus 9 U/mL (IQR 5.5-21) for patients without. For PR3-ANCA, median concentrations were 49 U/mL (IQR 19-129) for AAV patients compared to 8.4 U/mL (IQR 4.8-15) for non-AAV patients. *Abbreviations*: AAV (ANCA-associated vasculitis), ANCA (antineutrophil cytoplasmic antibodies), IQR (interquartile range), MPO (myeloperoxidase), PR3 (proteinase 3).

Overall, MPO- and PR3-ANCA concentrations were higher in AAV than in MPO/PR3-positive patients without AAV (*p* < 0.001). Median concentrations in AAV were 63 U/mL (IQR 25–210) for MPO-ANCA and 49 U/mL (IQR 19–129) for PR3-ANCA, compared to 9 U/mL (IQR 5.5–21) for MPO-ANCA and 8.4 U/mL (IQR 4.8–15) for PR3-ANCA in non-AAV patients (Fig 3). The lowest concentration in AAV patients for MPO-ANCA was 5.3 U/mL and for PR3-ANCA 3.3 U/mL; the highest concentration for MPO-ANCA was 1553 U/mL and for PR3-ANCA 2175 U/mL. Conversely, in ANCA-positive patients without AAV, the lowest concentration was 5.1 U/mL for MPO-ANCA and 3.1 for PR3-ANCA; the highest concentration in these patients was 81 U/mL for MPO-ANCA and 98 U/mL for PR3-ANCA.

### Comparison with previous studies

Table 4 provides a direct comparison between our results and the studies underlying the consensus guidelines, as well as other relevant prior studies.

**Table 4:** Comparison of ANCA testing strategies across studies highlighting the impact of disease prevalence and clinical context.

Study	Study design	Setting	Total N (AAV/ controls)	AAV prevalence	Testing strategy	PPV/ NPV	Key message	PMID	Link
**EUVAS total**	Multicentre diagnostic study	Tertiary centre	1175 (186 GPA, 65 MPA)	21%	IIF vs immunoassays	PPV ~ 90–95%, NPV ~ 93–97% (estimated)	High analytical accuracy (not real-world)	27481830	https://doi.org/10.1136/annrheumdis-2016-209507
**EUVAS – Copenhagen**	Cohort	Tertiary centre	283 (GPA 38, MPA 8)	16%	Same	High PPV expected	Selected population	idem	idem
**EUVAS – Leuven**	Cohort	Tertiary centre	301 (GPA 40, MPA 15)	18%	Same	High PPV expected	High-prevalence setting	idem	idem
**EUVAS – Maastricht**	Cohort	Tertiary centre	336 (GPA 68, MPA 16)	25%	Same	High PPV expected	Referral population	idem	idem
**EUVAS – Bad Bramstedt**	Case-control	Tertiary centre	255 (GPA 40, MPA 26)	N/A*	IIF vs immunoassays	High PPV expected	Enriched referral cohort	idem	idem
**Present study**	Retrospective cohort	Secondary care	Period 1: 4208 (80)	1,9%	Period 1: IIF → ELISA	Low PPV expected	Real-world testing strategy & interpretation	—	—
			Period 2: 481 (6)	1,2%	Period 2: IIF + ELISA				
			Period 3: 829 (21)	2,5%	Period 3: ELISA only				
**Other studies**	**Study design**	**Setting**	**Total N (AAV/ controls)**	**AAV prevalence**	**Testing strategy**	**PPV/ NPV**	**Key message**	**PMID**	**Link**
**Deka *et al.* 2021**	Retrospective cohort	Clinical (selected patients)	189 (suspected AAV)	Moderate	IIF vs ELISA	PPV 90.9%, NPV 92.2%	High specificity of ELISA in selected cohort	34602797	A Comparative Evaluation of Antigen-Specific Sandwich Immunoassay and Indirect Immunofluorescence Assay (IIF) in Detecting Antineutrophil Cytoplasmic Antibodies: Are We Ready to Replace IIF with ELISA as the Primary Screening Method? - PubMed
**Guchelaar *et al.* 2020**	Systematic review & meta-analysis	Mixed	Multiple studies	Variable	IIF vs immunoassays	Not directly reported	Confirms superior performance of immunoassays vs IIF	33197574	The value of anti-neutrophil cytoplasmic antibodies (ANCA) testing for the diagnosis of ANCA-associated vasculitis, a systematic review and meta-analysis – PubMed
**Badger *et al.* (JALM) 2025/2026**	Retrospective real-world lab study	Clinical laboratory	15,772 samples	Very low	Concurrent IIF + PR3 immunoassay	Not primary endpoint	IIF and immunoassays are complementary, supporting combination testing strategies.	not assigned yet	Concurrent Anti-PR3 Immunoassay and cANCA Indirect Immunofluorescence Testing Provide Complementary Information for Clinical Laboratory Detection of Antineutrophil Cytoplasmic Antibodies
**Loureiro-Amigo *et al.* 2026**	Retrospective cohort	Routine care	5157 (~47 AAV/ majority controls)	~1%	IIF + reflex immunoassay	PPV 6.1%, NPV 99.9%	PPV extremely low in unselected population	not assigned yet	https://doi.org/10.1093/rheumatology/keag072
**Aw *et al.* 2026 (gating)**	Retrospective cohort	Tertiary refferal lab	516 (19/ 497)	~3,8%	Unguided vs clinically gated testing	PPV 65,4%; NPV 99,6%; after implemention gating algoritm PPV 94,4%	Pre-test probability strongly determines the PPV of ANCA testing.	DOI: 10.1136/jcp-2026-210632	A classification criteria-based gating strategy improves anti-neutrophil cytoplasmic antibody (ANCA) testing performance characteristics for the diagnosis of ANCA-associated vasculitis | Journal of Clinical Pathology
**Consensus 2020**	Guideline	N/A	N/A	N/A	ELISA-first	N/A	Emphasizes selective testing	32663621	https://doi.org/10.1016/j.autrev.2020.102618

*In the Bad Bramstedt study, specific “cohorts of patients” with known diseases acted as a comparative control group, thereby more closely resembling a case-control design. For this reason prevalence was not calculated.

The multicentre EUVAS study formed the basis for the 2017 consensus. In three of the four tertiary referral centres in the study, cases were the patients tested for ANCA who had AAV. Disease controls were “consecutive patients who visited the respective hospital and for whom the medical doctor considered it important to request ANCA,” after which a diagnosis of AAV was excluded [10]. This is comparable to our study design. The Bad Bramstedt study, however, was different in design in that the controls were pre-existing cohorts of patients with specific inflammatory diseases. For this reason, we left the Bad Bramstedt study out of our prevalence calculations. The prevalence of AAV in the tested population of these tertiary centres was 16%, 18% and 25% respectively (calculated from S1 Table Damoiseaux *et al*. 2017). The average prevalence was 19.9%, roughly 10 times higher than the prevalence in our hospital. The high positive predictive value observed in the EUVAS study (~90–95%) reflects the relatively high prevalence of AAV in a selected tertiary care cohort. In contrast, in low-prevalence secondary care settings as shown in this study and other studies in Table 4, the PPV is expected to be substantially lower, emphasizing the importance of clinical pre-test probability and diagnostic strategy.

## Discussion

AAV is a multisystem disease that can manifest in virtually any organ system. The clinical distinction with other autoimmune diseases, malignancy and infectious diseases is often challenging, especially because these diseases may be accompanied by a positive ANCA test and secondary vasculitis [3]. Management of AAV relies on promptly starting immunosuppressive therapy and can differ significantly from the management of mimicking conditions. Therefore, it is important to differentiate between the various conditions correctly. Most important in this distinction are clinical presentation of the patient and clinical judgement. ANCA tests are important supporting tools: ANCA testing strategy and interpretation play an important role in the diagnostic process.

The pre-test probability of AAV depends on clinical presentation, but also on the prevalence of disease in the population. In clinical practice, ANCA tests are often ordered when the differential diagnosis is still broad. As AAV can have heterogeneous presentations, ANCA tests are frequently used to help ‘rule out’ AAV, instead of ‘ruling it in’ when there is already a high pre-test probability based on clinical presentation. We analysed different ANCA testing strategies throughout time in a large teaching hospital, in relation to the 1999 and 2017 consensus approaches. Our aim was to assess whether the 2017 consensus is equally applicable to secondary care centres with lower AAV prevalences, as to tertiary referral centres with a much higher prevalence in their tested population. [9,11]. This is important as most AAV patients are not diagnosed in expert centres [15].

Estimates of AAV prevalence in the general population are between 200 and 400 cases per million [16]. In our hospital population, 1.9% of patients for whom ANCA testing was requested had AAV. The 2017 consensus approach based its recommendations on a multicentre study including patients from tertiary referral centres; on average 19.9% of the tested patients had AAV – an approximately ten times higher percentage [10]. As shown in Fig 1 differences in prevalence impact the way a test result can be interpreted. Bossuyt *et al.* (2017) reported that regarding sensitivity and specificity, ELISA as first test performs equally to or better than IIF as screening test followed by confirmatory ELISA – ‘the 1999 ideal approach’ [11]. As PPV is highly dependent on disease prevalence, this may be a more important test parameter to look at than sensitivity and specificity, which are study population independent. Additionally, incorporating the use of likelihood ratios, which takes into account prior probabilities, to calculate the post-test probability, may be helpful in correctly interpretating ANCA test results. Unfortunately, reporting and using LRs is not common practice yet [17–19].

In our study period, 5518 first ANCA tests were requested in our centre. 286 (5.2%) samples were positive, but only 107 patients (37%) had AAV. The majority of ANCA-positive patients (63%) did not have AAV. ANCA-positivity in these patients was associated with the presence of infections, other autoimmune diseases, malignancies and use of certain drugs. These associations have been described extensively in the literature [3].

When regarding the different test strategies, i.e., following the 1999 consensus or the 2017 consensus, our results show that NPV and LR- are high throughout the periods, whichever test strategy is used. This illustrates that with a negative ANCA test result, irrespective of the testing strategy, chances of having AAV are low, making it suitable for *ruling out* AAV in most cases. As ELISA is a relatively easy test to perform, the recommendation of the 2017 consensus to first perform an ELISA is appropriate to exclude a diagnosis of AAV in the correct clinical context. Advantages of ELISA are lower costs and wider availability compared to IIF, as IIF interpretation is complex and requires specially trained personnel.

An important advantage of IIF is however that ANCA-IIF can be relevant in non-vasculitic autoimmune conditions such as autoimmune liver disease (AILD) and inflammatory bowel disease For example, in AILD, so-called atypical pANCA patterns (pANNA) are frequently observed. These antibodies are typically not directed against MPO or PR3, which may result in discordant findings between IIF and antigen-specific assays, underscoring the importance of interpreting ANCA results in their appropriate clinical context [3,20,21]. When the aim of the test is to confirm the diagnosis when there is (high) clinical suspicion of AAV, i.e., for *ruling in* AAV, our results show that ELISA as first test has a significantly better PPV and LR+ compared to IIF as first test. Using ELISA as first test is also suitable in this situation. However, a PPV of 63.6% means that a positive ELISA result is false positive in 36.4% of cases. This underlines that even though ELISA performed well as a first-line test in this real-world secondary care population, positive results should still be interpreted in combination with clinical presentation, antibody concentration, and, when appropriate, a second confirmatory test.

When IIF ánd ELISA are performed, PPV increases to 76.2 (*n.s.*) and specificity increases significantly from 98.5% to 99.5%. Performing two tests, where the second test is used to confirm a positive first test result, increases specificity and reduces false positive results. This is especially important in secondary care settings with lower AAV prevalence in the background population, as we have demonstrated that in fact the majority of ANCA-positive patients in our setting did not have AAV. Therefore, we believe it is important to consider a second test when there is low to moderate clinical suspicion of AAV and a positive first ANCA test.

Unfortunately, performing two tests does not overcome the problem of false-positivity entirely. Only ordering an ANCA test when it is appropriate and recognizing whether the aim is to *rule in* or *rule out* AAV, is essential. Possible ordering guidelines have been published and may reduce the rate of false-positive tests, without missing patients with AAV [22,23]. Integrating clinical judgement and ANCA testing, will enable physicians to correctly interpret test results leading to a correct diagnosis. Mimickers (Table 1) must always be carefully ruled out. Additionally, when there is no clear explanation for a positive ANCA test in non-AAV, it should be taken into account that ANCA-positivity can predate clinical AAV by years [24,25]. Follow-up for signs of vasculitis, including periodic urinalysis to detect glomerulonephritis early, should be considered. While in routine care true false positives related to other conditions are likely more common, this does not negate the need for cautious follow-up in selected cases. Finally, interference in the lab must be ruled out. This makes close communication between the clinician and laboratory expert essential.

The 2017 consensus approach only recommends performing a second test when there is high clinical suspicion of AAV and a negative first test, or if MPO- or PR3-ANCA concentrations are low. The former is important to avoid missing AAV cases. Regarding MPO/PR3 concentrations, our data show that these were generally higher in AAV patients than in MPO/PR3-positive patients without AAV. However, distribution was wide; low concentrations were seen in AAV while some MPO/PR3-positive patients without AAV had high concentrations. Additionally, concentrations may vary over time and potential treatment at time of first test may be of influence. For an individual patient, MPO/PR3 concentration alone cannot be used to determine if a patient has AAV or not, clinical context is key.

**Fig 4:**
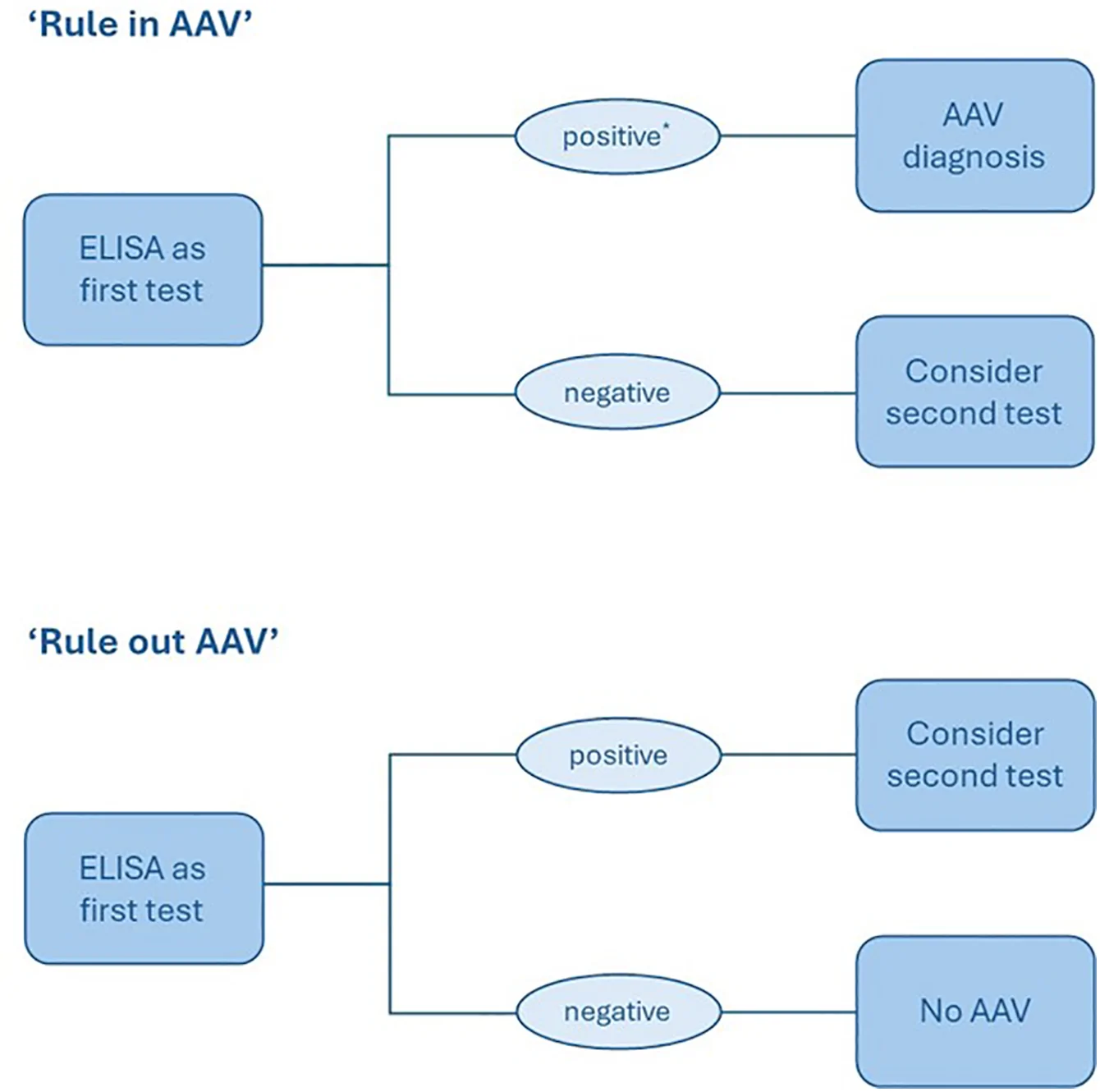
Proposed ANCA testing approach for secondary care settings. Our testing approach takes into consideration the objective of the clinician when requesting the test. In both situations, an ELISA can best be used as first test. When there is a high clinical suspicion of AAV and an ANCA test is requested to confirm the diagnosis (to ‘*rule in* AAV’), a positive test will support this. If the test is negative, a second test should be performed to avoid missing AAV cases. When an ANCA test is requested to ‘*rule out* AAV’ and exclude the diagnosis, a negative ELISA is sufficient. This is in accordance with the current recommendation. However, if clinical doubt exists about the diagnosis and the first test is positive, performing a second test (ELISA or IIF) can be considered, to increase specificity and positive predictive value of the testing strategy. In addition, other conditions that are associated with the presence of ANCA should be ruled out clinically. * In case of low antibody concentrations, consider a second test as well. *Abbreviations*: AAV (ANCA-associated vasculitis), ANCA (antineutrophil cytoplasmic antibodies), ELISA (enzyme-linked immunosorbent assay), IIF (indirect immunofluorescence).

Using our findings, we propose a testing approach tailored to secondary care settings, shown in Fig 4. When the aim is to *rule out* AAV when there is low clinical suspicion, an ELISA is usually sufficient. When the aim is to *rule in AAV*, i.e., confirm the diagnosis, a second test may be useful. This is the case when there is high clinical suspicion but a negative first test. Conversely, with a positive first test and diagnostic doubt, a second test should be considered as well, to minimize false positives and increase specificity and PPV. Test results should be interpreted carefully, in the context of clinical presentation, to optimize patient outcomes and healthcare efficiency.

Of note, no confirmatory tests were requested in period 3 in our centre, despite the comment accompanying the test result that in case of high clinical suspicion and negative ELISA, a second test should be considered. The reason a second test was never requested could be due to the fact that there was never a negative test in the case of high clinical suspicion of AAV, or because the consensus was not always followed. All 21 AAV cases in period 3 were MPO/PR3-positive by ELISA and during follow-up no new AAV cases were reported clinically, so we have no reason to believe guidelines were not followed correctly.

The 2017 consensus guidelines do not specify which type of second test should be ordered. Although our data do not allow us to compare all different combinations of tests, different approaches may be appropriate in different situations. If the aim of the ANCA test is to *rule in* AAV but a first antigen-specific test is negative, a second antigen-specific test (with comparable or higher sensitivity) is useful. If the aim is to *rule out* AAV, when a first antigen-specific assay is positive with a low concentration or unclear clinical context, IIF as a second test may be informative, because pattern information aids interpretation [26].

Our study has several limitations. A first limitation concerns sample size. While it is a strength that this study was long-term and monocentric – having a consistent pre-test probability and no differences in laboratory conditions – the sample size per period is not equal, which can make comparisons difficult.

We included only diagnostic tests, however, our search criterion was ‘first test performed at our hospital’. This means we cannot rule out that a first diagnostic test was performed elsewhere before referral to our hospital. This could possibly have influenced our lab results as these patients may have received immunosuppressants before the first ANCA test performed in our hospital. Both in the AAV-cohort and in the ANCA-positive patients without AAV, the use of immunosuppressants at the time of testing was considerable. Concluding, we report the performance of ANCA testing in over a decade in a large teaching hospital in the Netherlands and make a suggestion for a slightly modified testing approach tailored to secondary care settings. Current guidelines, developed largely from tertiary-care data, are of great value and remain the benchmark for practice. Our results provide complementary evidence from secondary care, where pre-test probability is typically lower. We believe our findings may inform future iterations of the guidelines, while also raising awareness of the limitations and appropriate use of ANCA testing in everyday clinical practice.

## Supporting information

S1 Table Dataset AAV, ANCA. (CSV)
